# Psychotropic Drugs for Older Adults With Psychiatric Disorders Presenting to the Emergency Department: Prescription Patterns and Treatment Outcomes

**DOI:** 10.1176/appi.prcp.20250002

**Published:** 2025-03-21

**Authors:** Marianna Mazza, Marcello Covino, Francesco Maria Lisci, Caterina Brisi, Georgios D. Kotzalidis, Giuseppe Marano, Francesca Abate, Maria Benedetta Anesini, Gianluca Boggio, Michele Ciliberto, Valeria De Masi, Cecilia Falsini, Ester Maria Marzo, Sara Rossi, Maria Chiara Spera, Alberto Torresi, Benedetta Simeoni, Francesco Franceschi, Gabriele Sani

**Affiliations:** ^1^ Unit of Psychiatry Fondazione Policlinico Universitario Agostino Gemelli IRCCS Rome Italy; ^2^ Department of Neuroscience Università Cattolica del Sacro Cuore Rome Italy; ^3^ Emergency Medicine Department Fondazione Policlinico Universitario Agostino Gemelli IRCCS Università Cattolica del Sacro Cuore Rome Italy

## Abstract

**Objective:**

A high proportion of patients presenting to Emergency Departments (EDs) consists of vulnerable elderly people. Mental illnesses are frequently related to advancing age, with difficult to detect symptoms. The aim of this study was to investigate psychiatric drug use by geriatric patients in an ED for whom a psychiatric consultation was needed, according to their psychopathology and severity of outcome.

**Methods:**

During 2014 to 2023, 342 aged patients (35.96% men and 64.04% women; median age = 73 years, range 68–79 years) sought help at the ED of our hospital and required psychiatric consultation.

**Results:**

Male sex, nonsuicidal self‐harm, and access through emergency medical services were predictors of >48‐h ED stay/hospital admission (the main severity outcome). Patients with bipolar disorder (BD) and psychotic disorders were more socially disadvantaged and with significantly longer length of stay at the ED than the other groups. Additionally, BD patients had more psychiatric drug prescriptions at discharge than patients in the other diagnostic groups. Current nonsuicidal self‐harm emerged as a predictor of >48‐h ED stay/hospital admission.

**Conclusions:**

Future studies should focus on investigating suicide risk in ED contexts, on correct diagnosing and on prescription appropriateness of medications in older adults with psychiatric disorders referring to the ED so to tailor‐cut treatment on individual patients' needs.

**Relevant to Clinical Practice:**

This study highlights the significant impact of psychiatric conditions on healthcare utilization among elderly patients presenting to the ED. The findings emphasize the need for improved diagnosis and management of psychiatric disorders, medication optimization, and tailored treatment approaches.

## Highlights

- What is the primary question addressed by this study? Investigation on psychotropic drugs prescriptions and treatment outcomes of geriatric patients in an Emergency Department (ED) for whom a psychiatric consultation was needed.
- What is the main finding of this study? Male sex and bipolar/psychotic diagnoses were associated with worse outcome. Nonsuicidal self‐harm predicted longer ED stay and hospitalization and bipolar disorder was associated with higher psychopharmacological prescription rates.
- What is the meaning of the finding? Older patients with psychiatric disorders who refer to the ED need a careful management and a tailored care specifically adapted to clinical and psychopathological characteristics.

Life expectancy in the world population is increasing, hence elderly persons represent an increased proportion of patients seen in clinical practice and presenting to Emergency Departments (EDs). According to the World Health Organization, approximately 14% of adults aged 60 and over live with a mental disorder, and mental disorders among older adults account for 10.6% of the total years lived with disability for this age group (1). The COVID‐19 pandemic negatively impacted psychosocial wellbeing, especially among vulnerable older individuals, and individuals with multimorbidity and mental health comorbidities have an increased likelihood of using unplanned secondary care including ED visits and emergency hospitalizations (2).

Mental illnesses are frequently related to advancing age, with symptoms that are usually difficult to detect. Furthermore, many psychiatric manifestations, such as depression or anxiety, represent not only severe problems but also exacerbate or even hide other comorbidities (3).

A study conducted among elderly Europeans found that half of individuals aged 65–84 years had experienced at least one mental disorder in their lifetime; the most prevalent disorders were anxiety disorders, followed by mood and substance‐related disorders (4). Risk factors for the development of any mental disorder were being unmarried, without religious affiliation, with more physical illnesses and a lower quality of life (5).

There is an extensive use of medications in elderly patients, particularly psychoactive drugs, although there are limited practice guidelines for aged people and there is little knowledge of age‐related physiological changes affecting most of the pharmacokinetic processes in the body (6). The particular pharmacokinetics and pharmacodynamics of old age and the use of polypharmacy due to the prevalence of multimorbidity tend to dangerously amplify the risk of drug interactions and untoward side effects (7, 8).

Many geriatric patients are referred to hospital psychiatric consultation services. In England and Scotland there is a trend towards increase year‐by‐year in the request of psychiatric liaison consultations by EDs (9). In the ED, geriatric patients with acute psychiatric symptoms are often treated with psychotropic drugs (10). Lack of knowledge and expertise to adequately manage older people in the ED leads to poor patient outcomes, such as mortality and functional decline. Addressing the multifaceted needs of older people (medical, psychological, social, functional, and environmental) remains a clinical practice priority (11). This proves to be particularly pivotal for geriatric patients with psychiatric disorders.

Among patients accessing the ED, about 3% are eventually hospitalized in a psychiatric department (12). ED‐related outcomes vary according to the study; for example, the effectiveness of intervention is measured through ED visit reduction, less time spent in ED (length‐of‐stay [LOS]), decreased ED wait times, and clinical outcomes such as mortality, hospitalization, healthcare system use, and costs (13). Other times, outcomes like mortality may be parceled into in‐hospital and 30‐day mortality and examined along 30‐day rehospitalization and LOS (14). In elderly patients (≥65 years of age), to differentiate groups of patients destined to psychiatric community residence *vs*. those living in their own home after visiting an ED, mortality, return visits to the ED, hospitalization 30‐day after ED visit, hospitalization within the same year of the index episode may be used as outcomes (15). Patients with mental health problems referring to ED have also been classified according to the frequency of service use as Single ED users, Repeat ED users, High ED users, and Very high and recurrent high ED users; in this case, risk of patient hospitalization and mortality were chosen as outcomes (16).

The aim of this study was to investigate psychiatric drug use by geriatric patients in an ED for whom a psychiatric consultation was needed, according to their psychopathology and severity of outcome, distinguishing between patients discharged or referred to other services within 2 days (“mild” or control group) and patients being held for at least 2 days at the ED or subsequently hospitalized (“severe” group). We also aimed at identifying predictors of hospitalization and prolonged ED admission (>48 h).

## METHODS

### Study Population

This is a single‐center, retrospective study conducted in a large university hospital, Fondazione Policlinico Universitario Agostino Gemelli IRCCS, located in Rome. This hospital has an annual ED attendance of ≈75,000 patients with more than 87% of patients being adults (17). We included patients over the age of 65 who were consecutively admitted to the ED from January 1, 2014 to June 30, 2023 and who needed psychiatric consultation. For patients with more than one hospitalization during the above period, we included data of the first hospitalization only. We split our population study into two samples, one consisting of patients eventually hospitalized and those who were discharged after more than 48 h at the emergency room (the “severe” group), the other of controls, consisting of patients who stayed for <48 h at the ED and were discharged without needing hospitalization (the “mild,” control group).

### Study Variables

We examined the electronic medical records of included patients, collecting their demographic and clinical data, patterns of ED access, diagnostic group assigned by the consultant, psychotropic treatment received, and events occurring during the hospital stay, including the outcome at discharge. For the purpose of this study we noted age, sex, and triage level; the latter is classified in Italy as Red color code, meaning emergency, immediate life threat, absolute priority, to be visited immediately; Yellow code, that is, urgency, serious injury, maximum effort to reduce patient waiting time; Green code, that is, minor urgency, apparently not life threatening, intervention possibly deferred; and White code, no urgency, apparently not serious, visit carried out when possible, compatibly with all other emergencies. We combined the latter two codes into one group (Table 1).

**TABLE 1:** Demographic and clinical characteristics of included patients. Categorical values are expressed as counts (%), continuous variables are expressed as medians (interquartile range).

	All cases (*n* = 334)	Anxiety disorders (*n* = 34)	SSOPD (*n* = 38)	BD (*n* = 71)	MDD (*n* = 231)	*p*‐value
Clinical picture at ED access						
Age in years (range)	73 (69–79)	74 (70–79)	70 (67–76)	71 (68–78)	73 (69–80)	0.067
Sex, male (percent)	140 (37.4%)	10 (29.4%)	12 (31.6%)	29 (40.8%)	89 (38.9%)	0.580
Access at the emergency medical service, *n* (%)	161 (43%)	13 (38.2%)	19 (50%)	34 (47.9%)	95 (41.7%)	0.550
Triage level						
Red code, *n* (%)	59 (15.8%)	4 (11.8%)	7 (18.4%)	12 (16.9%)	36 (15.6%)	0.581
Yellow code, *n* (%)	135 (36.1%)	9 (26.5%)	13 (34.2%)	22 (31%)	91 (39.4%)	
Combined Green and White codes, *n* (%)	180 (48.1%)	21 (61.8%)	18 (47.4%)	37 (52.1%)	104 (45%)	
CGI‐S, median (range)	4 (3–5)	3 (2–4)	5 (4–6)	4 (3–5)	4 (3–5)	**<0.001**
Current non‐suicidal self‐harm, *n* (%)	62 (16.6%)	2 (5.9%)	6 (15.8%)	16 (22.5%)	38 (16.5%)	0.199
Delirium, *n* (%)	25 (6.7%)	1 (2.9%)	4 (10.5%)	1 (1.4%)	19 (8.2%)	0.127
Somatic symptom disorder, *n* (%)	16 (4.3%)	3 (8.8%)	‐	2 (2.8%)	11 (4.8%)	0.27
Psychomotor agitation, *n* (%)	81 (21.8%)	5 (14.7%)	8 (21.1%)	21 (29.6%)	47 (20.5%)	0.289
Acute medical comorbidities, *n* (%)	132 (35.3%)	11 (32.4%)	9 (23.7%)	22 (31%)	90 (39%)	0.230
Psychiatric history						
Previous psychiatric diagnosis, *n* (%)	249 (66.6%)	17 (50.0%)	30 (78.9%)	56 (78.9%)	146 (63.2%)	**0.005**
Previous psychiatric treatments, *n* (%)	243 (65%)	17 (50%)	29 (76.3%)	62 (87.3%)	135 (58.4%)	**<0.001**
Previous psychiatric hospitalizations, *n* (%)	39 (10.4%)	1 (2.9%)	12 (31.6%)	16 (22.5%)	10 (4.3%)	**<0.001**
Family history of psychiatric disorders, *n* (%)	15 (4.0%)	1 (2.9%)	0	6 (8.5%)	8 (3.8%)	0.138
New‐onset disorder, *n* (%)	59 (15.8%)	8 (23.5%)	3 (7.9%)	7 (9.9%)	41 (17.7%)	0.119
Substance use disorder, *n* (%)	26 (7.0%)	1 (2.9%)	3 (7.9%)	6 (8.5%)	16 (6.9%)	0.766
Currently prescribed psychiatric drugs, median (range)	1 (0–2)	1 (0–1)	2 (1–3)	1 (0–2)	1 (0–2)	0.244
Socio‐economic conditions						
Living alone, *n* (%)	51 (13.6%)	4 (11.8%)	4 (10.5%)	8 (11.3%)	35 (15.2%)	0.750
Living with his/her family, *n* (%)	135 (36.1%)	11 (32.4%)	11 (28.9%)	25 (35.2%)	88 (38.1%)	0.689
Homeless, *n* (%)	2 (0.5%)	‐	2 (5.3%)	‐	‐	**<0.001**
Assisted by legal or social guardian, *n* (%)	5 (1.4%)	‐	1 (2.6%)	3 (4.2%)	1 (0.4%)	0.240
Disability living allowance, *n* (%)	42 (11.2%)	‐	8 (21.1%)	13 (18.3%)	21 (9.1%)	**0.006**
ED interventions, outcomes						
Total LOS in ED (days), median (range)	1.2 (0.3–5.7)	0.3 (0.2–2.3)	2.4 (0.6–7.3)	1.6 (0.6–5.4)	1.13 (0.3–6)	**<0.001**
Need for >1 psychiatric consultation in ED	121 (32.4%)	3 (8.8%)	14 (36.8%)	32 (45.1%)	72 (31.2%)	**0.002**
Observation >48 h	155 (41.4%)	9 (26.5%)	22 (57.9%)	33 (46.5%)	91 (39.4%)	**0.036**
Psychiatric drugs prescribed at discharge from ED	2 (1–3)	1 (1–2)	2 (1–3)	3 (2–3)	2 (1–3)	**<0.001**
Therapy modification after consultation	233 (62.3%)	24 (70.6%)	24 (63.2%)	44 (62.0%)	141 (61.0%)	0.761
Discharged from the ED to an outpatient service	203 (54.3%)	26 (76.5%)	14 (36.8%)	37 (52.1%)	126 (54.5%)	**0.011**

*Note*: Significant differences marked by bold characters.

Abbreviations: BD, bipolar disorder; CGI‐S, Clinical Global Impressions‐Severity; ED, Emergency Department; LOS, length‐of‐stay; MDD, major depressive disorder; SSOPD, schizophrenia spectrum and other psychotic disorders.

The consulting psychiatrist classified cases according to the following categories: anxiety, psychosis, bipolar disorder (BD), and unipolar depression (major depressive disorder [MDD]). He/she completed the Clinical Global Impressions scale‐Severity (CGI‐S), a clinician rated scale with a single item asking the clinician the degree of average mental illness of the patient during the last week based on reported symptoms, behavior and function and rated 1–7 on a Likert scale, where 1 is normal, not at all ill, 2 is borderline mentally ill, 3 is mildly ill, 4 is moderately ill, 5 is markedly ill, 6 is severely ill, and 7 is among the most extremely ill patients (18). The psychiatrist recorded also non‐suicidal self‐harm acts, the presence of delirium, somatic symptom disorder, psychomotor agitation, and acute medical comorbidities. Suicidal ideation was assessed through direct questions during the interview. Furthermore, the consultant investigated and annotated the patient's psychiatric family history, and his/her socio‐economic status (subdivided in living alone, living with his/her family, homelessness, assisted by a legal or social guardian, and disability living allowance). We extracted data from retrieved records to determine whether treatment was modified after the consultation, whether prescriptions at discharge included psychiatric drugs, and whether there was need for more than one psychiatric consultation for the same patient.

### Outcome Measures

The endpoints were the identification of predictors of the need for hospitalization and prolonged (>48 h) ED stay. We also focused on background differences between the severe and the mild groups of patients who needed psychiatric consultations.

### Ethics

The study was carried out adhering to the Principles of Human Rights, as were adopted by the World Medical Association at the 18th WMA General Assembly, Helsinki, Finland, June 1964, further amended by the 64th WMA General Assembly, Fortaleza, Ceará, Brazil, in October 2013. It received approval from the Fondazione Policlinico Agostino Gemelli Protocol N°: 0025817/22, ID:5121 of 03/08/2022.

### Statistical Analysis

We first assessed the normality of distribution of the chosen variables in the severe and mild groups by carrying out the Shapiro–Wilk normality test (19). The categorical variables were presented as numbers and percentages. The continuous normally distributed variables were presented as the mean ± standard deviation; the non‐normally distributed data were presented as the median (inter‐quartile range), and the binary or ordinal variables were presented as absolute frequency (%). We conducted parametric tests for continuous variables with a normal distribution (Student's *t* test) and non‐parametric tests for those with non‐normal distribution (Mann–Whitney *U*‐test). The LOS was calculated from the time of ED admission to the discharge or death. Predictors were entered in multivariate analysis to find out if the significant results were maintained. A two‐sided *p*‐value of <0.05 was set as the significance level. All data were analyzed through the SPSS v26® (IBM, 2018).

## RESULTS

During the nine‐and‐a‐half–year period we considered, 2378 aged patients (1033 men [43.44%], 1345 women [56.56%]) sought help at the ED of our hospital. Of these, 386 required psychiatric consultation (16.32%). Of these, 12 patients were excluded from the analysis due to lack of data, thus leaving a total sample of 374 patients (median age = 73 years, range 68–79 years) of whom 140 were men (37.43%) and 234 women (62.57%). Of these patients, 32 refused hospitalization or abandoned the ED voluntarily; their data were excluded from the final data analysis, thus leaving a final sample of 342 patients, of whom 123 were men (35.96%) and 219 women (64.04%). The median age and age range remained the same as above.

### Background Differences Between Psychiatric Diagnoses

Patients with BD and psychotic disorders had significantly more family psychiatric history, previous psychiatric treatments and psychiatric hospitalizations than patients with MDD or anxiety disorders (Table 1). Patients with psychosis showed significantly more homelessness than all other groups, and these patients along with patients with BD had significantly more Disability Living Allowance than the MDD and anxiety disorders groups. Patients with psychosis and BD had significantly more LOS in ED than the other groups, while patients with BD had more need for more than one psychiatric consultation and more need for staying at ED for more than 48 h than patients in the other groups. BD patients had more psychiatric drug prescriptions at discharge than patients in the other diagnostic groups, while patients with anxiety disorders were referred at discharge to outpatient services (Table 1). The groups did not differ on other variables.

### Predictors of the Need for Hospital Admission

Male sex and access through the emergency medical services were predictors of admission to ward (Table 2). Both resisted to the multivariate analysis. In the multivariate analysis, psychosis and MDD predicted prolonged ED stay or hospital admission, as did severity assessed through the CGI‐S.

**TABLE 2:** Predictors of the need for hospital admission (*N* = 342).

	Discharged from the ED (*N* = 203)	Admitted to hospital (*N* = 139)	*p* value	Multivariate OR (95% CI)	Multivariate *p* value
Age in years, median (range)	73 (69–79)	73 (69–79)	0.909		
Sex, male (percent)	57 (28.1%)	66 (47.5%)	<0.001	2.56 (1.57–4.23)	**<0.001**
Access by EMS	66 (32.5%)	83 (59.7%)	<0.001	2.93 (1.72–4.86)	**<0.001**
Triage level					
Red code	20 (9.9%)	35 (25.2%)	<0.001		
Yellow code	73 (36%)	50 (36%)			
Green and White codes combined	110 (54.2%)	54 (38.8%)			
Main diagnosis					
Anxiety disorders	26 (12.8%)	4 (2.9%)	<0.001	*Reference category*	
SSOPD	14 (6.9%)	22 (15.8%)		7.08 (1.85–27.1)	**0.004**
BD	37 (18.2%)	27 (19.4%)		3.48 (1.00–12.1)	0.050
MDD	126 (62.1%)	86 (61.9%)		4.25 (1.32–13.7)	**0.015**
CGI	3 (3–4)	4 (3–5)	<0.001	1.43 (1.18–1.73)	**<0**.**001**
Current non‐suicidal self‐harm	18 (8.9%)	31 (22.3%)	<0.001	1.13 (0.55–2.31)	0.735
Delirium	12 (5.9%)	12 (8.6%)	0.330		
Somatic symptom disorder	9 (4.4%)	6 (4.3%)	0.959		
Psychomotor agitation	45 (22.3%)	28 (20.3%)	0.660		
Comorbidities	1 (0–2)	1 (0–2)	0.870		
Acute medical comorbidities	76 (37.4%)	50 (36%)	0.760		
Psychiatric history					
Previous psychiatric diagnosis	138 (68%)	93 (66.9%)	0.830		
Previous psychiatric treatments	133 (65.5%)	91 (65.5%)	0.992		
Previous psychiatric hospitalizations	15 (7.4%)	19 (13.7%)	0.570		
Family history of psychiatric disorders	6 (3.0%)	9 (6.5%)	0.119		
New onset disorder	29 (14.3%)	25 (18.0%)	0.357		
Substance use disorder	9 (4.4%)	11 (7.9%)	0.178		
Currently prescribed psychiatric drugs, *n* (range)	1 (1–2)	1 (0–2)	0.125		
Socio‐economic conditions					
Living alone	31 (15.3%)	14 (10.1%)	0.162		
Living with his family	74 (36.5%)	49 (35.3%)	0.820		
Homeless	0	2 (1.4%)	0.164		
Assisted by a legal or social guardian	1 (0.4%)	4 (2.9%)	0.163		
Disability living allowance	19 (9.4%)	21 (15.1%)	0.104		
ED interventions, outcomes					
Therapy modification after consultation	140 (69.0%)	79 (56.8%)	0.022	0.70 (0.43–1.16)	0.169
Need for >1 psychiatric consultation in ED	58 (28.6%)	52 (37.4%)	0.086		

*Note*: Thirty‐two patients who refused hospitalization or spontaneously abandoned the ED were excluded from data analysis. Triage was excluded from the multivariate models due to its covariation with the CGI‐S value. Significant results in bold characters.

Abbreviations: BD, bipolar disorder; CGI‐S, Clinical Global Impressions‐Severity; CI, confidence interval; ED, Emergency Department; EMS, emergency medical services; MDD, major depressive disorder; OR, odds ratio; SSOPD, schizophrenia spectrum and other psychotic disorders.

### Predictors of the Need for the Combined Endpoint Prolonged ED Stay (>48 h) or Hospital Admission

Male sex and access through the emergency medical services were predictors of prolonged ED stay or hospital admission. However, only access through the emergency medical services resisted to the multivariate analysis (Table 3). In the multivariate analysis, psychosis predicted prolonged ED stay or hospital admission, as severity as assessed through the CGI‐S and current non‐suicidal self‐harm.

**TABLE 3:** Predictors of the need for the combined endpoint prolonged ED stay (>48 h) or hospital admission (*N* = 374).*

	ED stay <48 h (*N* = 201)	Prolonged ED stay or ward admission (*N* = 173)	*p* value	Multivariate OR (95% CI)	Multivariate *p* value
Age in years, median (range)	73 (68–79)	73 (69–78.5)	0.848		
Sex, male, *n* (percent)	66 (32.8%)	74 (42.8%)	0.048	1.57 (0.99–2.50)	0.055
Access by EMS, *n* (percent)	66 (32.5%)	83 (59.7%)	<0.001	3.08 (1.94–4.90)	**<0.001**
Triage					
Red code	10 (5.0%)	49 (28.3%)	<0.001		
Yellow code	71 (35.3%)	64 (37.0%)			
Green and White codes combined	120 (59.7%)	60 (34.7%)			
Main diagnosis					
Anxiety disorders	25 (12.4%)	9 (5.2%)	0.001	*Reference category*	
SSOPD	11 (5.5%)	27 (15.6%)		4.62 (1.48–14.4)	**0.009**
BD	35 (17.4%)	36 (20.8%)		1.89 (0.70–5.02)	0.209
MDD	130 (64.7%)	101 (58.4%)		1.89 (0.70–5.02)	0.181
CGI‐S	3 (3–4)	4 (3–5)	<0.001	1.29 (1.08–1.51)	**0.005**
Current non‐suicidal self‐harm	18 (8.9%)	31 (22.3%)	<0.001	1.98 (1.01–3.92)	**0.049**
Delirium	12 (6.0%)	13 (7.5%)	0.551		
Somatic symptom disorder	9 (4.5%)	7 (4.0%)	0.837		
Psychomotor agitation	48 (23.9%)	33 (19.1%)	0.261		
Comorbidities (*n*)	1 (0–2)	1 (0–2)	0.870		
Acute medical comorbidities	66 (32.8%)	66 (38.2%)	0.284		
Psychiatric history					
Previous psychiatric diagnosis	138 (68%)	93 (66.9%)	0.830		
Previous psychiatric treatments	133 (65.5%)	91 (65.5%)	0.992		
Previous psychiatric hospitalizations	14 (7.0%)	25 (14.5%)	0.018	1.26 (0.57–2.79)	0.568
Family history of psychiatric disorders	5 (2.5%)	10 (5.8%)	0.106		
New‐onset disorder	29 (14.4%)	30 (17.3%)	0.441		
Substance use disorder	13 (6.5%)	13 (7.5%)	0.691		
Currently prescribed psychiatric drugs (*n*)	1 (0–2)	1 (0–2)	0.308		
Socio‐economic conditions					
Living alone	30 (14.9%)	21 (12.1%)	0.434		
Living with his family	72 (35.8%)	63 (36.4%)	0.905		
Homeless	0	2 (1.2%)	0.126		
Assisted by a legal or social guardian	1 (0.5%)	4 (2.3%)	0.128		
Disability living allowance	18 (9.0%)	24 (13.9%)	0.133		
ED interventions, outcomes					
Therapy modification after consultation	131 (65.2%)	102 (59.0%)	0.216		
Psychiatric drugs prescribed at discharge from ED, *n* (range)	2 (1–3)	2 (1–3)	0.341		
Need for >1 psychiatric consultations in ED	45 (22.4%)	76 (43.9%)	<0.001		

*Note*: * Comprises patients who abandoned the ED or refused hospitalization. Significant results in bold characters.

Abbreviations: BD, bipolar disorder; CGI‐S, Clinical Global Impressions‐Severity; CI, confidence interval; ED, Emergency Department; EMS, emergency medical services; MDD, major depressive disorder; OR, odds ratio; SSOPD, schizophrenia spectrum and other psychotic disorders.

### Psychopharmacological Treatments of Patients in ED Who Needed Psychiatric Consultation

Patients for whom an increase in existing psychotropic drug treatment was required, those who were added a new psychotropic drug and those receiving a psychotropic drug did not differ significantly among the various diagnostic groups. However, patients with anxiety disorders required a change in their psychotropic drug prescription were significantly more than patients with unipolar depressive disorder and patients with psychosis, while patients with BD required significantly less changes in their psychiatric drugs (Table 4). SSRIs were significantly more prescribed to patients with MDD, lithium and valproate significantly more to patients with BD, first‐generation antipsychotics (FGAs) and second‐generation antipsychotics (SGAs) significantly more to patients with psychosis compared to BD, who in turn received more FGA and SGA prescriptions than patients with MDD or anxiety disorders.

**TABLE 4:** Psychopharmacological treatments of patients in ED who needed psychiatric consultation.

	All (*n* = 334)	Anxiety disorders (*n* = 34)	SSOPD (*n* = 38)	BD (*n* = 71)	MDD (*n* = 231)	*p*
Increase existing PDT	198 (52.9%)	19 (55.9%)	19 (50%)	42 (59.2%)	118 (51.1%)	0.64
Change PDT	54 (14.4%)	10 (29.4%)	5 (13.2%)	4 (5.6%)	35 (15.2%)	**0.013**
Add new PDT	227 (60.7%)	20 (58.8%)	23 (60.5%)	38 (53.5%)	146 (63.2%)	0.53
Patients taking PDT	257 (68.7%)	23 (67.6%)	29 (76.3%)	49 (69%)	156 (67.5%)	0.75
Delirium	25 (6.7%)	1 (2.9%)	4 (10.5%)	1 (1.4%)	19 (8.2%)	0.127
BDZ	136 (36.4%)	15 (44.1%)	9 (23.7%)	22 (31%)	90 (39%)	0.166
TCA	12 (3.2%)	2 (5.9%)	‐	5 (7%)	5 (2.2%)	0.102
SSRI	77 (20.6%)	5 (14.7%)	7 (18.4%)	7 (9.9%)	58 (25.1%)	**0.033**
SNRI	24 (6.4%)	1 (2.9%)	‐	6 (8.5%)	17 (7.4%)	0.24
Trazodone	16 (4.3%)	1 (2.9%)	‐	2 (2.8%)	13 (5.6%)	0.35
Mirtazapine	16 (4.3%)	2 (5.9%)	2 (5.3%)	1 (1.4%)	11 (4.8%)	0.601
Vortioxetine	7 (1.9%)	‐	‐	‐	7 (3%)	0.22
Bupropion	2 (0.5%)	‐	‐	‐	2 (0.9%)	0.74
Lithium	13 (3.5%)	‐	‐	12 (16.9%)	1 (0.4%)	**0.000**
Valproic acid	34 (9.1%)	‐	10 (26.3%)	12 (16.9%)	12 (5.2%)	**0.000**
Lamotrigine	6 (1.6%)	1 (2.9%)	2 (5.3%)	2 (2.8%)	1 (0.4%)	0.099
Oxcarbazepine	4 (1.1%)	‐	1 (2.6%)	2 (2.8%)	1 (0.4%)	0.24
Carbamazepine	1 (0.3%)	‐	‐	1 (1.4%)	‐	0.23
Topiramate	1 (0.3%)	‐	‐	‐	1 (0.4%)	0.89
Gabapentin	19 (5.1%)	‐	2 (5.3%)	5 (7%)	12 (5.2%)	0.49
Pregabalin	13 (3.5%)	‐	1 (2.6%)	3 (4.2%)	9 (3.9%)	0.67
FGAs	39 (10.4%)	2 (5.9%)	9 (23.7%)	10 (14.1%)	18 (7.8%)	**0.014**
SGAs	74 (19.8%)	7 (20.6%)	16 (42.1%)	15 (21.1%)	36 (15.6%)	**0.002**
Z‐drugs	21 (94.4%)	‐	3 (7.9%)	3 (4.2%)	15 (6.5%)	0.39

*Note*: Significant results in bold characters.

Abbreviations: BD, bipolar disorder; BDZ, benzodiazepines; ED, Emergency Department; FGAs, first‐generation antipsychotics; MDD, major depressive disorder; PDT, psychotropic drug treatment; SGAs, second‐generation antipsychotics; SSOPD, schizophrenia spectrum and other psychotic disorders; SSRI, selective serotonin reuptake inhibitors; SNRI, serotonin–norepinephrine reuptake inhibitors; TCA, tricyclic antidepressants; Z‐drugs, non‐benzodiazepine hypnotics.

**FIGURE 1:**
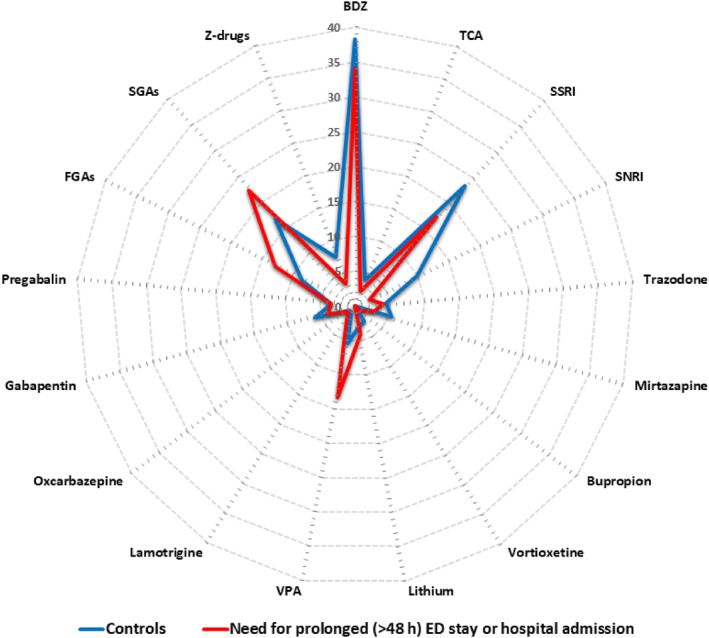
Radar chart of psychiatric drug prescription pattern in the combined group of hospitalized patients or those discharged after >48 h at the emergency room (red) compared to controls (i.e., patients not needing prolonged stay at ED and discharged or referred to outpatient services) (blue). BDZ, benzodiazepines; ED, emergency department; FGAs, first‐generation antipsychotics; SGAs, second‐generation antipsychotics; SNRI, serotonin‐noradrenaline reuptake inhibitors; SSRI, selective serotonin reuptake inhibitors; TCA, tricyclic antidepressants; VPA, valproate, valproic acid; Z‐drugs, non‐benzodiazepine hypnotics.

The distribution of individual psychotropic drugs and drug classes across the four diagnostic groups is shown in Figure S1. The pattern of psychotropic drug prescription grossly overlapped in the two outcome groups, severe and mild, with patients in the combined group of hospitalized patients or those discharged after >48 h at the emergency room differing for being prescribed more valproate, as shown by the radar chart (Figure 1).

Deaths in ED occurred for 10 of all 374 included patients (2.67%); no deaths occurred in the anxiety disorders and the psychosis subgroups, while 3 patients died in the bipolar group (4.2%) and 7 in the MDD group (3%). The difference was not statistically significant (*p* = 0.432). Suicidal ideation was reported by 30 (8%) of all patients. There were no cases of suicidal ideation in the 35‐patient anxiety disorders subgroup (0%); 10 patients (14.1%) in the 71‐patient bipolar subgroup reported suicidal thinking, 5 (13.2%) in the 38‐patient psychosis subgroup, and 15 (6.5%) in the 231‐patient MDD subgroup; pooling the two higher suicidal ideation groups, the difference versus MDD was statistically significant (*χ* ^2^ = 4.8625; *p* = 0.027). However, by performing Fisher's exact test, no difference emerged between the four diagnostic subgroups (*p* = 0.948). No death occurred by suicide.

## CONCLUSIONS

In this study we found 16% of aged patients who referred to the ED of a large general Roman hospital in need of a psychiatric consultation. About two‐thirds of these patients were women and one‐third men; this 1.78:1 women/men ratio was higher than the one of all patients attending an ED (1.30:1), indicating that the female gender could represent a risk factor for needing a psychiatric consultation while visiting the ED. However, it was the male gender that constituted a predictor of subsequent hospitalization. This finding is generally in line with previous data in the literature. Studies have shown that female gender is often associated with a higher likelihood of seeking psychiatric consultations in EDs, particularly for anxiety and stress‐related disorders. On the other hand, male gender has been identified as a predictor of subsequent hospitalization, especially in cases involving substance use disorders and more severe mental health issues (20).

Being treated for psychosis or BD predicted prolonged stay at the ED or subsequent hospitalization. Prescriptions matched diagnoses, with antidepressants prescribed for cases diagnosed with MDD, mood stabilizers for BD, and antipsychotics for psychoses. As expected and reflected in population statistics, the larger diagnostic groups were the depressive and anxiety disorders, followed by BD and psychosis (21, 22, 23).

Although there were no deaths by suicide, suicidal thinking was prominent in the bipolar and psychosis subgroups, more than double the rate found in the MDD subgroup, despite the lack of statistical significance. This is in line with the literature that reports suicidal ideation as a major reason for seeking help at an ED (9, 24, 25).

ED overcrowding is an increasing public health issue, and it has been noticed that during the last decade, the overall number of ED visits has continuously increased worldwide and this was expected, since the world population continues to increase. If the number of EDs does not increase proportionally, and this is what happens due to a race towards funding cuttings to health services, overcrowding ensues. Older patients with psychiatric manifestations are overcrowding‐vulnerable ED patients (26). Patients with mental health problems may visit the ED for various reasons, that is, to gain prompt access to mental health services, to get reassured about their condition, to manipulate their significant others (16). Healthcare providers in ED have often limited time and knowledge, hence limited opportunities to provide optimal care for patients with mental health problems; as a consequence, these patients are at risk of poor hospital experiences and treatment outcomes (27).

To investigate psychiatric drug use by geriatric patients in an ED for whom a psychiatric consultation was needed and to identify predictors of hospitalization and prolonged ED admission we chose two outcomes. One, which was the more severe one, consisted in the patient either kept at the ED for more than 48 h or admitted to a hospital ward, while the other, milder, included patients who were discharged within 48 h or referred to outpatient services. Patients with BD and psychotic disorders were more socially disadvantaged and showed significantly LOS at the ED than the other groups. Additionally, BD patients had more psychiatric drug prescriptions at discharge than patients in the other diagnostic groups. This is in line with previous studies showing that patients with BD and psychotic disorders seeking help at the ED had lower household income and high recurrence of acute symptoms (28, 29, 30, 31, 32, 33). In fact, a relationship has been shown between poverty and mental health problems (34, 35).

Disease severity, as assessed through the CGI‐S, predicted prolonged ED stay or hospital admission, that is, the more severe outcome, particularly in patients with psychosis. Rating disease severity in the ED is important for achieving prompt and effective treatment, since the first clinical judgment can predict prognosis and clinical course and is essential for timely treatment (36). Our results seem to confirm the assumption that clinical severity and clinical instability, measured through the CGI, are independent and robust predictors of future risk of hospitalization in psychiatric patients, across diagnoses, age groups, and genders (37).

In our sample, male sex and access through the emergency medical services were predictors of >48 h ED stay/hospital admission. It has previously been described that older patients with more severe presenting complaints at triage, who arrive by ambulance/police, with longer LOS, and with mood and psychotic disorders are more likely to require hospital admission (38). In patients with psychiatric disorders, gender disparities in the frequency of hospitalization and psychotropic prescription have been reported, but there is still debate and a lack of gender‐specific literature on how to best stratify ED patients for risk (39).

In our study current non‐suicidal self‐harm emerged as a predictor of >48 h ED stay/hospital admission. As already observed, the higher hospitalization and death rates found among older ED patients with non‐suicidal self‐injury than among suicide attempters indicate that lethal, intentional, self‐destructive behaviors in late life may occur, even in the absence of suicidal intent (40). It is also known that non‐fatal self‐harm is one of the most frequent reasons for emergency hospital admission and the strongest risk factor for subsequent suicide (41). Older patients are a particularly vulnerable population at risk for neurocognitive disorders, social exclusion, functional disability, physical conditions, bereavement, and may be overwhelmed by feelings of loneliness and hopelessness (42). Access to ED should be provided timely for all patients presenting with self‐harm; these patients should undergo comprehensive psycho‐social assessment, and should be followed up closely in association with community mental health services. It is important that older adults who harm themselves without explicit suicidal intent are assessed by mental health specialists trained to carefully evaluate risks and needs in this group of individuals, as they have complex co‐morbidities which can have an impact on their treatment (43).

Regarding psychotropic treatment, we found that patients requiring a change in their psychotropic drug prescription belonged to the anxiety disorder subgroup significantly more than patients in the unipolar depressive disorder and psychosis subgroups. Since anxiety is frequently associated with acute somatic symptoms, patients with anxiety disorders tend towards high health care service utilization and frequently refer to EDs (44). The prevalence of anxiety increases in older patients, and ED stay may be particularly distressing (45). These patients, more than those in the other diagnostic groups, undergo changes in their prescriptions during ED stay, since they are more demanding and induce clinicians to attempt finding the right drug prescription, balancing risks and benefits. In our sample, BD patients required the least changes in their psychiatric drug prescriptions. We may presume that being the effective mood stabilizers less numerous than the available antipsychotics, antidepressants and anti‐anxiety agents, there are less choices and less probability for changes. Treatment of the elderly population with BD is challenging, because drug metabolism may change with advancing age, and dramatic pharmacokinetic differences result from changes in adipose tissue, free water, protein binding, and drug distribution (46). Monotherapy should be the ultimate goal in treating elderly patients, but BD may require the addition to lithium or valproate or other efficacious antiepileptic drugs of atypical antipsychotics.

Limitations of this study include the relatively small sample and the heterogeneity of diagnoses and administered drugs, even when we pool them in drug classes (including the fact that many patients received multiple psychotropic drug prescriptions); furthermore, this was a single‐center study, and its results cannot be extended to other populations. However, the study has been conducted in one of the largest Italian hospitals, with a considerable attendance of patients covering a great metropolitan area which at least is representative of the country's capital. Our real‐world data may highlight demographic and clinical characteristics of patients over the age of 65 admitted to the ED over 10 years who needed psychiatric consultation. Furthermore, clinicians are required to complete patient information on the electronic clinical information system during each patient's visit; hence, this procedure minimizes missing data. Since randomization was not possible due to the retrospective nature of the study, we adjusted all our results for potential confounders by using a multivariable regression model.

This study showed that older patients with BD and psychotic disorders were more socially disadvantaged and stayed longer at the ED than other groups. Additionally, BD patients had more psychiatric drug prescriptions at discharge than patients in other diagnostic groups. In our sample, male sex and access through emergency medical services predicted >48 h ED stay/hospital admission. Current non‐suicidal self‐harm emerged as a predictor of >48 h ED stay/hospital admission. We need to improve our knowledge about clinical characteristics and medication management among older patients with psychiatric disorders who refer to the ED, so to avoid inappropriate prescriptions, improve safety and quality of assistance, and recommend a tailored care specifically adapted to these patients' needs.

## Supporting information

Figure S1
