# Supplementary material for: Psychotropic Drugs for Older Adults With Psychiatric Disorders Presenting to the Emergency Department: Prescription Patterns and Treatment Outcomes
Source: Psychiatr Res Clin Pract. 2025 Mar 21;7(2):117–27. doi: 10.1176/appi.prcp.20250002 (PMC12178202; doi:10.1176/appi.prcp.20250002)
Supplement: Supplementary file 1 — Figure S1 [file RCP2-7-117-s001.docx]

*
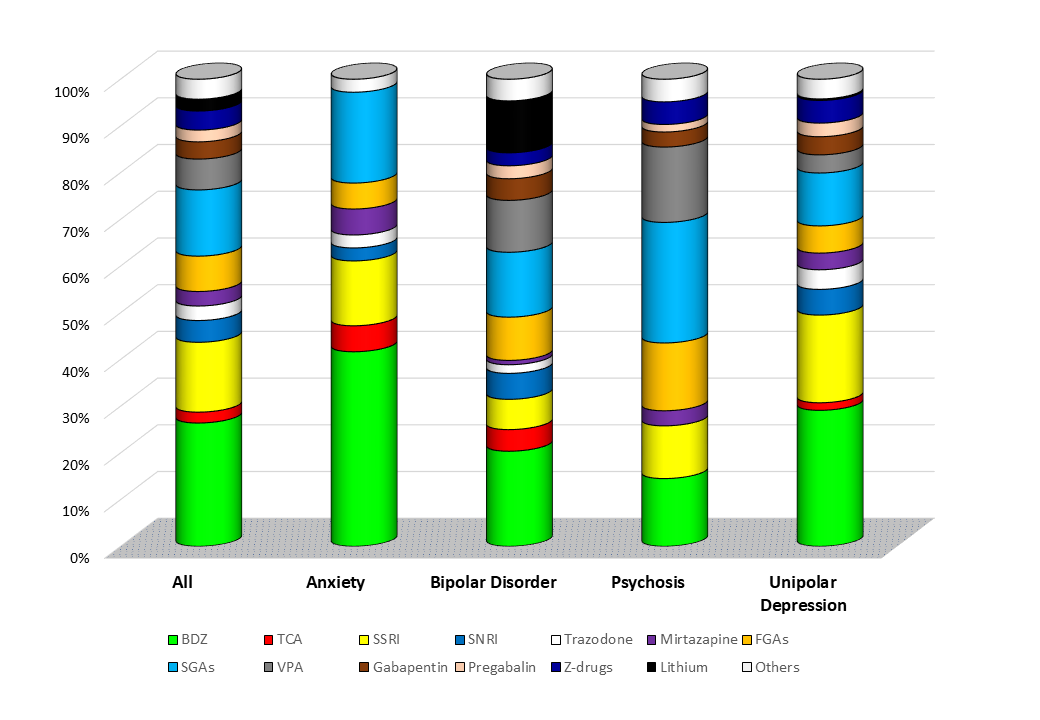
*

Figure *S*1. Main psychotropic drug prescriptions in the study population.

Note: BDZ, benzodiazepines; FGAs, first-generation antipsychotics; SGAs, second-generation antipsychotics; SNRI, serotonin-noradrenaline reuptake inhibitors; SSRI, selective serotonin reuptake inhibitors; TCA, tricyclic antidepressants; VPA, valproate, valproic acid (valproate); Z-drugs, non-benzodiazepine hypnotics.
